# Chrysoeriol suppresses hyperproliferation of rheumatoid arthritis fibroblast-like synoviocytes and inhibits JAK2/STAT3 signaling

**DOI:** 10.1186/s12906-022-03553-w

**Published:** 2022-03-16

**Authors:** Jia-Ying Wu, Ying-Jie Chen, Xiu-Qiong Fu, Jun-Kui Li, Ji-Yao Chou, Cheng-Le Yin, Jing-Xuan Bai, Ying Wu, Xiao-Qi Wang, Amy Sze-man Li, Lut Yi Wong, Zhi-Ling Yu

**Affiliations:** 1 Research and Development Centre for Natural Health Products, HKBU Shenzhen Institute of Research and Continuing Education, Shenzhen, China; 2 School of Chinese Medicine, Consun Chinese Medicines Research Centre for Renal Diseases, Hong Kong Baptist University, Hong Kong, China; 3 Centre for Cancer and Inflammation Research, School of Chinese Medicine, Hong Kong Baptist University, Kowloon Tong, Hong Kong, China; 4 JaneClare Transdermal TCM Therapy Laboratory, Hong Kong Baptist University, Hong Kong, China

**Keywords:** Apoptosis, Chrysoeriol, JAK2, STAT3 signaling, Proliferation, Rheumatoid arthritis fibroblast-like synoviocytes

## Abstract

**Background:**

Fibroblast-like synoviocytes (FLS) have cancer cell-like characteristics, such as abnormal proliferation and resistance to apoptosis, and play a pathogenic role in rheumatoid arthritis (RA). Hyperproliferation of RA-FLS that can be triggered by the activation of interleukin-6/signal transducer and activator of transcription 3 (IL-6/STAT3) signaling destructs cartilage and bone in RA patients. Chrysoeriol is a flavone found in medicinal herbs such as Chrysanthemi Indici Flos (the dried capitulum of *Chrysanthemum indicum* L.). These herbs are commonly used in treating RA. Chrysoeriol has been shown to exert anti-inflammatory effects and inhibit STAT3 signaling in our previous studies. This study aimed to determine whether chrysoeriol inhibits hyperproliferation of RA-FLS, and whether inhibiting STAT3 signaling is one of the underlying mechanisms.

**Methods:**

IL-6/soluble IL-6 receptor (IL-6/sIL-6R)-stimulated RA-FLS were used to evaluate the effects of chrysoeriol. CCK-8 assay and crystal violet staining were used to examine cell proliferation. Annexin V-FITC/PI double staining was used to detect cell apoptosis. Western blotting was employed to determine protein levels.

**Results:**

Chrysoeriol suppressed hyperproliferation of, and evoked apoptosis in, IL-6/sIL-6R-stimulated RA-FLS. The apoptotic effect of chrysoeriol was verified by its ability to cleave caspase-3 and caspase-9. Mechanistic studies revealed that chrysoeriol inhibited activation/phosphorylation of Janus kinase 2 (JAK2, Tyr1007/1008) and STAT3 (Tyr705); decreased STAT3 nuclear level and down-regulated protein levels of Bcl-2 and Mcl-1 that are transcriptionally regulated by STAT3. Over-activation of STAT3 significantly diminished anti-proliferative effects of chrysoeriol in IL-6/sIL-6R-stimulated RA-FLS.

**Conclusions:**

We for the first time demonstrated that chrysoeriol suppresses hyperproliferation of RA-FLS, and suppression of JAK2/STAT3 signaling contributes to the underlying mechanisms. This study provides pharmacological and chemical justifications for the traditional use of chrysoeriol-containing herbs in treating RA, and provides a pharmacological basis for developing chrysoeriol into a novel anti-RA agent.

**Supplementary Information:**

The online version contains supplementary material available at 10.1186/s12906-022-03553-w.

## Background

Rheumatoid arthritis (RA) is a chronic inflammatory disease that affects 0.5–1.0% of the global population. RA causes joint damage, disability and other comorbidities, seriously degrading the patients’ quality of life [1, 2]. Fibroblast-like synoviocytes (FLS) have cancer cell-like characteristics, such as abnormal proliferation and resistance to apoptosis, and play a pathogenic role in RA [3]. Hyperproliferation of RA-FLS leads to the damage of bones and cartilage of RA patients. Activation of signal transducer and activator of transcription 3 (STAT3) promotes proliferation and suppresses apoptosis of RA-FLS [4]. High level of interleukin-6 (IL-6), commonly found in inflamed joints of RA patients, causes abnormal activation/phosphorylation of STAT3 (Tyr705) [5]. Therefore, the IL-6/STAT3 pathway has been regarded as a target for treating RA.

Disease-modifying antirheumatic drugs (DMARDs), including conventional DMARDs (e.g., methotrexate and leflunomide), targeted DMARDs (e.g., Janus kinase inhibitors) and biological DMARDs (e.g., tumor necrosis factor inhibitors, IL-6 inhibitors, IL-1 inhibitors and T-cell immunomodulators), are the mainstay for treating RA [6]. DMARDs can efficiently ameliorate joint swelling and pain of RA patients [7]. However, they have severe adverse effects that have restricted their clinical use. The most severe side effect of DMARDs is infection, a result of their inhibitory effects on the host immune system [8]. Stomach upset, hepatotoxicity, blood dyscrasias and interstitial lung disease are commonly associated with DMARDs as well [9]. Other available anti-RA drugs, including non-steroidal anti-inflammatory drugs (NSAIDs) and glucocorticoids, also have side effects, including cardiovascular, upper gastrointestinal, metabolic and endocrine effects [10, 11]. Developing safe and effective natural products as novel anti-inflammatory agents is a hot research topic in anti-RA drug discovery.

Chrysoeriol (CSR) is a flavonoid compound found in diverse medicinal herbs such as Cardiospermi Halicacabi Herba (all parts of *Cardiospermum halicacabum* L.) and Chrysanthemi Indici Flos (the dried capitulum of *Chrysanthemum indicum* L.) [12, 13]. These herbs are commonly used for treating RA and have been shown to have anti-arthritic effects in animal models [14, 15]. These facts suggest that CSR has anti-arthritic effects. In a previous study, we found that CSR ameliorates 12-O-tetradecanoylphorbol-13-acetate (TPA)-induced acute skin inflammation in mice. Moreover, in the animal model and in RAW264.7 cells, CSR inhibits STAT3 signaling [16]. Whether CSR inhibits proliferation of, and induces apoptosis in RA-FLS is unknown.

In this work, we investigated the effects of CSR on hyperproliferation of IL-6/sIL-6R-stimulated RA-FLS and explored the involvement of the JAK2/STAT3 pathway in the effects.

## Materials and methods

### Reagents

CSR (purity > 99%, determined by HPLC) was purchased from Extrasynthese (Genay Cedex, France). Indomethacin, crystal violet, phosphate‐buffered saline (PBS) and dimethylsulfoxide (DMSO) were purchased from Sigma Chemical Co. (St. Louis, MO, USA). CCK-8 (Cell Counting Kit-8) was purchased from TransGen Biotech (Beijing, China). Recombinant human IL-6 and recombinant human soluble IL-6 receptor α (sIL-6R) were purchased from PeproTech (Rocky Hill, NY, USA). Janus kinase 2 (JAK2, ab39636) and phospho-JAK2 (Tyr1007/1008, ab32101) monoclonal antibodies, HRP-conjugated secondary antibodies (ab7090, ab97040), and Annexin V-FITC Apoptosis Staining / Detection Kit (#ab14085) were purchased from Abcam (Cambridge, CB2 0AX, UK). B-cell lymphoma 2 (Bcl-2, sc-7382) and GAPDH (sc-365062) monoclonal antibodies were obtained from Santa Cruz Biotechnology (Santa Cruz, CA, USA). Myeloid cell leukemia 1 (Mcl-1, #94296), caspase-9 (#9508), caspase-3 (#9662), STAT3 (#12640), phospho-STAT3 (Tyr705, #9131) and lamin B1 (#12586) monoclonal antibodies were obtained from Cell Signaling Technology (Boston, MA, USA). All other chemicals used were purchased from Sigma and were of analytical grade.

### Cell culture

RA-FLS were purchased from Cell Applications (San Diego, CA). L929 and MIHA cells were purchased from American Type Culture Collection (ATCC; Rockville, MD, USA). Fetal bovine serum (FBS) and Dulbecco’s Modified Eagle Medium (DMEM) were purchased from HyClone (Logan UT, USA). Cells were maintained in high glucose DMEM containing 10% FBS and 1% penicillin/streptomycin (P/S, GIBCO, USA) at 37 ℃ in a humidified atmosphere of 5% CO_2_. CSR and indomethacin were dissolved in DMSO at the concentrations of 40 mM and 100 mM, respectively. IL-6 was reconstituted in distilled water to 50 µg/ml and sIL-6R was reconstituted in PBS to 50 µg/ml (PH 7.2). Solutions for cell assays were filtered with syringe filters (0.22 μm) and stored at -20 ℃. The stock solutions were diluted with DMEM immediately before experiments. The final concentration of DMSO in cell culture was 0.1%.

### Cell viability assay

CCK-8 assay was performed to determine the cytotoxicity of CSR on RA-FLS, MIHA and L929 cells. Cells (3 × 10^3^/well) in 200 μl DMEM were seeded in 96-well plates, incubated at 37 ℃ for 24 h and given a fresh medium change. The cells were treated with various concentrations of CSR (0, 5, 10, 20, 40, 80 μM) or indomethacin (100 μM) for 1 h and then stimulated with IL-6/sIL-6R (100 ng/ml each) for 24 h or 48 h. Cells in the control group were treated with solvents (DMSO for CSR; PBS for IL-6/sIL-6R). In each well, 20 μl of CCK-8 solution was added and then incubated for 4 h. Absorbance of each well was measured at 450 nm with a microplate spectrophotometer (BD Biosciences, USA).

### Crystal violet staining

The crystal violet staining assay was employed to visualize the effects of CSR on the proliferation of RA-FLS. Cells (1 × 10^6^ cell/dish) seeded in 60 mm dishes were treated with CSR at various concentrations (0, 10, 20 μM) for 1 h and stimulated with IL-6/sIL-6R (100 ng/ml each) for 48 h. Afterwards, cells were fixed with 10% formalin for 5 min, followed by staining with 0.05% crystal violet solution in distilled water for 30 min. Cells were then washed and photographed. Crystal violet was solubilized with 1 ml of 33% glacial acetic acid. Absorbance was measured at 570 nm using a microplate spectrophotometer.

### Apoptosis assay

Apoptotic effects of CSR on RA-FLS were measured by Annexin V-FITC/PI double staining using an Apoptosis Detection Kit following the manufacturer’s protocol. Cells (1 × 10^5^ cell/well) were seeded in 6-well plates overnight and then treated as described in the Crystal violet staining section. Both detached and adherent cells were harvested and then incubated in 100 μl labeling solution (5 µl of Annexin V-FITC, 5 µl of PI, 10 µl of 10 × binding buffer and 80 µl of H_2_O) in darkness at room temperature for 15 min. After that, 400 µl of 1 × binding buffer was added to stop the staining reaction. Flow cytometric analysis was then performed with a BD Accuri C6 flow cytometer (BD Biosciences, USA). In the flow cytometric scatter graphs, cells undergoing apoptosis are shown in the upper-right quadrant (late-stage apoptotic cells) and the lower-right quadrant (early-stage apoptotic cells).

### Immunoblotting

Lysates were prepared from cultured RA-FLS as previously described [17]. Cell nuclear and cytoplasmic extracts were prepared using the Mammalian Nuclear and Cytoplasmic Protein Extraction Kit (TransGen Biotech, Beijing, China) following the manufacturer’s protocol. Protein concentrations were measured using a Quick Start™ Bradford Protein Assay (Bio-Rad, USA).

Western blot assays were performed as previously described [16]. Immunoreactive bands were visualized using the Enhanced Chemiluminescence Detection Kit (Invitrogen, USA). Image J software was used to measure the intensity of each band. The relative level of each protein of interest was normalized to the endogenous β-actin, GAPDH or lamin B1 in each experiment.

### Adenoviral transduction

Adenovirus expressing green fluorescent protein (GFP)-flag-tagged STAT3C (Ad-STAT3C) and control adenovirus expressing GFP (Ad-Empty vector) were purchased from Vigene Biosciences (Shandong, China). STAT3C (A662C, N664C mutant) is a constitutively activated variant of STAT3. RA-FLS (6 × 10^5^ cells/dish) were seeded in 100 mm dishes and transduced with Ad-STAT3C (7.8 × 10^7^ pfu/ml, RA-FLS^STAT3C^) or Ad-Empty vector (7.8 × 10^7^ pfu/ml, RA-FLS^Empty vector^) for 24 h, and then supernatant was discarded and replaced with fresh medium. After 12 h, transduced RA-FLS were used for experiments.

### Statistical analysis

Data are presented as the means ± standard deviation (SD). Comparison of quantitative data in multiple groups was performed using one-way analysis of variance (ANOVA) followed by Tukey's test. *P* < 0.05 was regarded as statistically significant.

## Results

### CSR suppressed hyperproliferation of IL-6/sIL-6R-stimulated RA-FLS

**Fig. 1:**
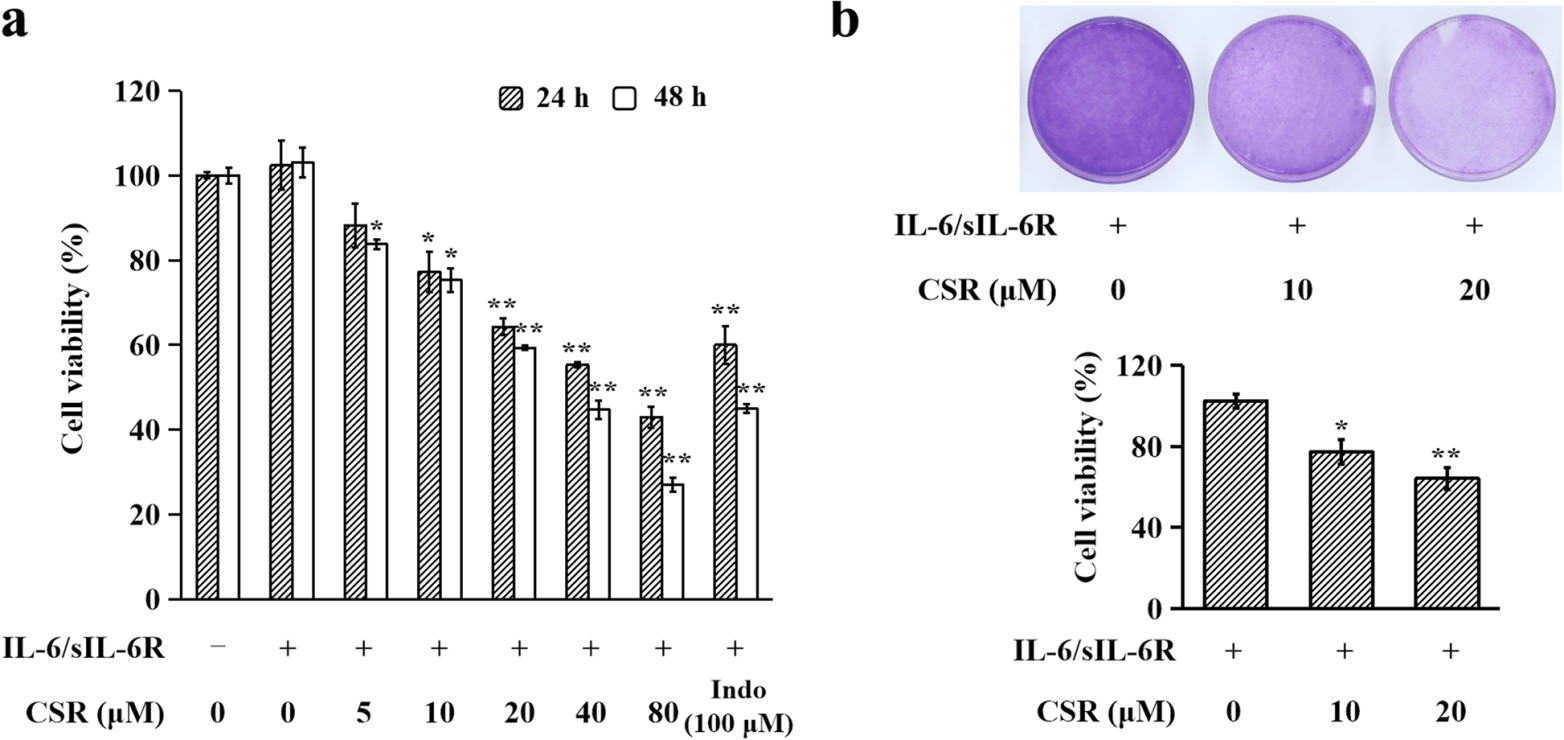
CSR suppressed hyperproliferation of IL-6/sIL-6R-stimulated RA-FLS. (**a**) Cell viability determined using CCK-8 assays. Viability of IL-6/sIL-6R plus CSR solvent-treated cells was regarded as 100%. Data from three independent experiments are presented as mean ± SD. * *P* < 0.05, ** *P* < 0.01 *vs.* IL-6/sIL-6R plus CSR solvent-treated group of corresponding treatment duration. (**b**) Cell proliferation determined using crystal violet staining. Representative photographs of viable cells are shown in the upper panel and quantitative results are shown in the lower panel. Data are expressed as mean ± SD of 3 independent experiments. * *P* < 0.05, ** *P* < 0.01 *vs.* IL-6/sIL-6R plus CSR solvent-treated group

CCK-8 assay was used to evaluate the effect of CSR on hyperproliferation of IL-6/sIL-6R-stimulated RA-FLS. Results showed that pre-treatments with CSR (5, 10, 20, 40, 80 μM; 24 or 48 h) reduced the viability of IL-6/sIL-6R (100 ng/ml each)-stimulated RA-FLS in time- and dose-dependent manners (Fig. 1a). In agreement with previous reports, the positive control drug indomethacin also suppressed the hyperproliferation of IL-6/sIL-6R-stimulated RA-FLS [18]. The anti-proliferative effect of 100 μM indomethacin against IL-6/sIL-6R-stimulated RA-FLS was comparable to that of 40 μM CSR. Crystal violet staining confirmed that CSR had dose-dependent and significant inhibitory effects on the proliferation of IL-6/sIL-6R-stimulated RA-FLS (Fig. 1b). Anti-proliferative activities of CSR were also evaluated in normal human liver-derived MIHA cells and in mouse fibroblast L929 cells. Viabilities of IL-6/sIL-6R (100 ng/ml each)-stimulated MIHA and L929 cells were not significantly affected by a 24-h incubation with up to 40 μM CSR (Additional file 1). These findings indicate that CSR suppresses hyperproliferation of RA-FLS and has no obvious influence on the proliferation of normal cells.

### CSR induced apoptosis in IL-6/sIL-6R-stimulated RA-FLS

**Fig. 2:**
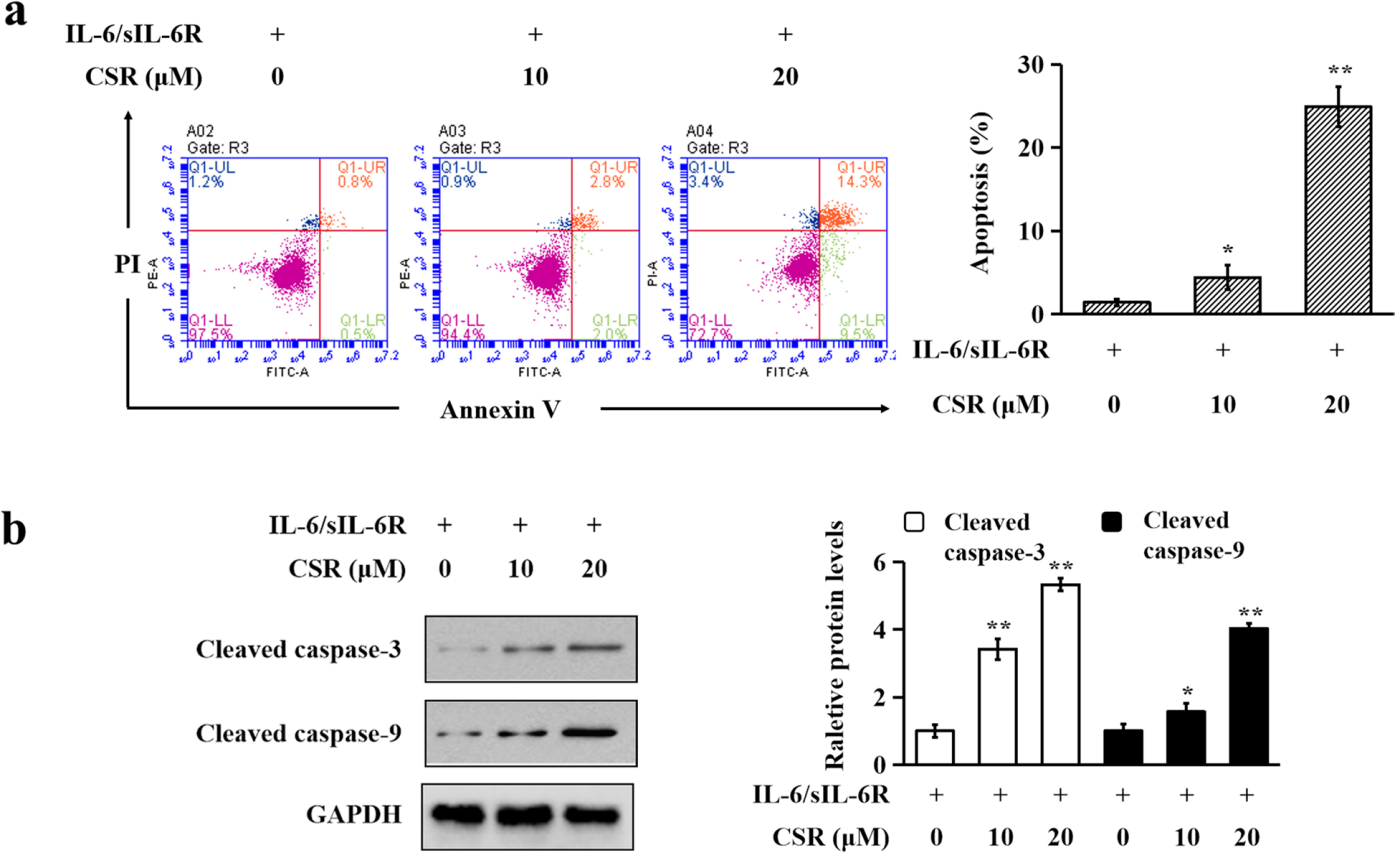
CSR induced apoptosis in IL-6/sIL-6R-stimulated RA-FLS. (**a**) Representative flow cytometric scatter graphs are shown in the left panel. Apoptotic cell percentages are shown in the right panel. (**b**) Immunoblotting analyses of cleaved caspase-3 and cleaved caspase-9 are shown. RA-FLS were incubated with indicated concentrations of CSR for 1 h and then stimulated with IL-6/sIL-6R (100 ng/ml each) for 24 h. GAPDH served as the loading control. Representative immunoblotting results (the left panel) and quantitative results (the right panel) are shown. Each membrane was cut based on molecular weights of proteins to be examined before hybridisation with primary antibodies. Original images of all blots are presented in Additional file 3. Data are expressed as mean ± SD of 3 independent experiments. * *P* < 0.05, ** *P* < 0.01 *vs.* IL-6/sIL-6R plus CSR solvent-treated group

Flow cytometry assay was used to determine the pro-apoptotic effects of CSR on IL-6/sIL-6R-stimulated RA-FLS after Annexin V-FITC/PI double staining. As shown in Fig. 2a, CSR treatments induced cell apoptosis. After a 48-h treatment, apoptosis rates in the control group, low dose CSR group (10 μM) and high dose CSR group (20 μM) were 1.4 ± 0.4%, 4.4 ± 1.5% and 24.9 ± 2.9%, respectively. CSR also significantly and dose-dependently elevated protein levels of cleaved caspase-3 and cleaved caspase-9 (Fig. 2b), two apoptotic markers, confirming its apoptotic effects in this cell model.

### CSR inhibited the JAK2/STAT3 pathway in IL-6/sIL-6R-stimulated RA-FLS

**Fig. 3:**
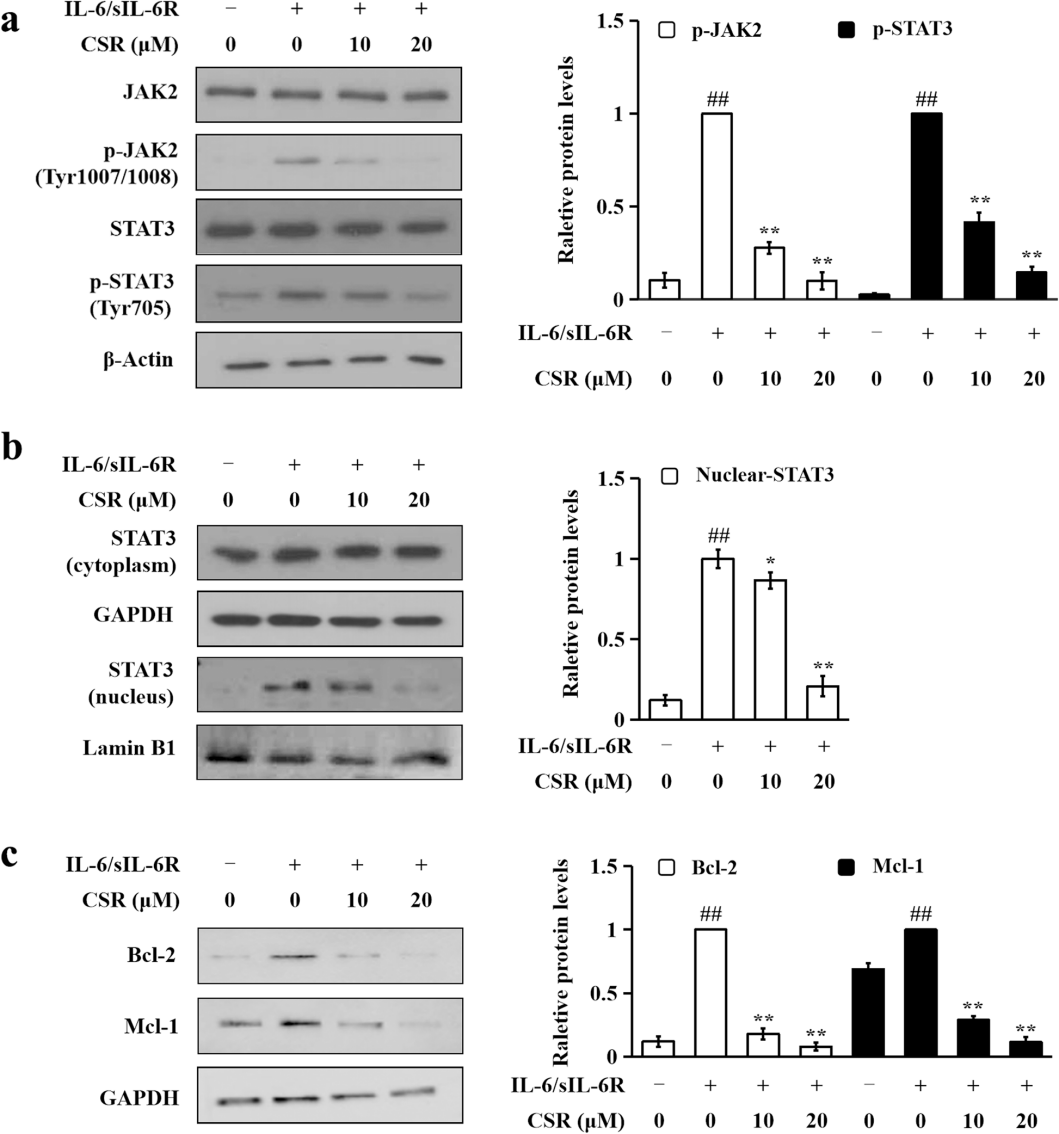
CSR inhibited the JAK2/STAT3 pathway in IL-6/sIL-6R-stimulated RA-FLS. Cells were incubated with indicated concentrations of CSR for 1 h and then stimulated with IL-6/sIL-6R (100 ng/ml each) for 40 min. (**a**) Protein levels of JAK2, phospho-JAK2 (Tyr1007/1008), STAT3 and phospho-STAT3 (Tyr705). β-Actin served as the loading control. (**b**) Protein levels of STAT3 in cytoplasmic and nuclear extracts. GAPDH and lamin B1 served as loading controls of cytoplasmic and nuclear extracts, respectively. (**c**) Protein levels of Bcl-2 and Mcl-1. GAPDH served as the loading control. In (**a**) – (**c**), representative immunoblotting results are shown in left panels. Quantitative results are shown in right panels. Each membrane was cut based on molecular weights of proteins to be examined before hybridisation with primary antibodies. Original images of all blots are presented in Additional file 4. Data are expressed as mean ± SD of 3 independent experiments. * *P* < 0.05, ** *P* < 0.01 *vs.* IL-6/sIL-6R plus CSR solvent-treated group. ## *P* < 0.01 *vs.* solvents-treated group

As shown in Additional file 2, among 3 tested stimulation durations (20, 40, 80 min) with IL-6/sIL-6R (100 ng/ml each), 40-min stimulation induced the greatest activation/phosphorylation of STAT3 (Tyr705) in RA-FLS. Thus, RA-FLS stimulated with IL-6/sIL-6R (100 ng/ml each) for 40-min were used as the cell model in the subsequent assays. In the present study, we found that pre-treatments of CSR for 1 h dose-dependently inhibited IL-6/sIL-6R-induced phosphorylation of JAK2 (Tyr1007/1008) and STAT3 (Tyr705) without affecting the total JAK2 and STAT3 levels (Fig. 3a). Figure 3b shows that CSR dose-dependently and significantly decreased the nuclear STAT3 level in IL-6/sIL-6R-stimulated RA-FLS. IL-6/sIL-6R alone or in combination with CSR did not affect the protein level of STAT3 in the cytoplasm. As shown in Fig. 3c, IL-6/sIL-6R upregulated protein levels of Bcl-2 and Mcl-1, which are STAT3 target genes involved in cell survival, while CSR dose-dependently and significantly suppressed the upregulation. These findings indicate that CSR inhibits the JAK2/STAT3 pathway in IL-6/sIL-6R-stimulated RA-FLS.

### Over-activation of STAT3 attenuated the inhibitory effect of CSR on RA-FLS hyperproliferation

**Fig. 4:**
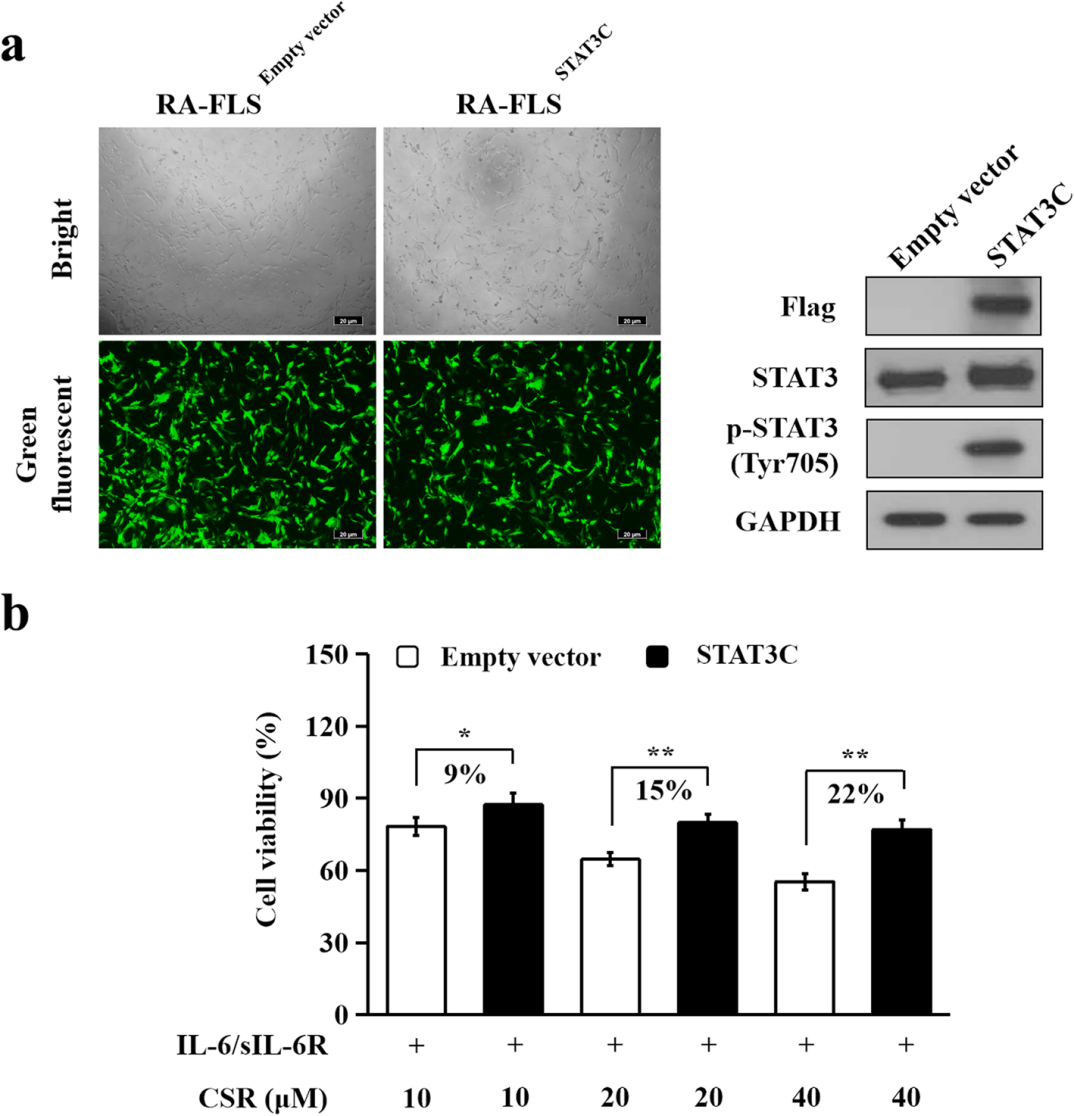
Over-activation of STAT3 attenuated the inhibitory effects of CSR on RA-FLS hyperproliferation. RA-FLS were transduced with Ad-STAT3C (RA-FLS^STAT3C^) or Ad-Empty (RA-FLS^Empty vector^) plasmid. (**a**) Green fluorescent protein (GFP) expression and the protein levels of phospho-STAT3 (Tyr 705), STAT3 and Flag in RA-FLS^Empty vector^ and RA-FLS^STAT3C^. Cells expressing GFP displayed green fluorescence. Representative green fluorescent and bright-field microscopy images of RA-FLS^Empty vector^ and RA-FLS^STAT3C^ are shown in the left panel (Scale bar: 10 μm). Representative immunoblotting results are shown in the right panel. Each membrane was cut based on molecular weights of proteins to be examined before hybridisation with primary antibodies. Original images of all blots are presented in Additional file 5. (**b**) Over-activation of STAT3 attenuated the inhibitory effect of CSR on RA-FLS hyperproliferation. Transduced RA-FLS were incubated with indicated concentrations of CSR for 1 h and then stimulated with IL-6/sIL-6R (100 ng/ml each) for 48 h. Cell viability was assessed using CCK-8 assays. Data are expressed as mean ± SD of 3 independent experiments. Differences of relative cell viabilities between CSR-treated RA-FLS^Empty vector^ and CSR-treated RA-FLS^STAT3C^ were calculated. * *P* < 0.05, ** *P* < 0.01

To determine whether STAT3 signaling contributed to the inhibitory effects of CSR on the viability of RA-FLS, we over-activated STAT3 in RA-FLS by transducing STAT3C into the cells. Green fluorescent and bright-field microscopy images of RA-FLS showed that the transduction was successful (Fig. 4a). Immunoblotting showed that transduction with Ad-STAT3C in RA-FLS caused remarkable elevation of phosphorylated STAT3 (Tyr705) level compared to transduction with Ad-Empty vector (Fig. 4a), showing an overactivation of STAT3 in RA-FLS. Upon STAT3 overactivation, the inhibitory effects of 10, 20 and 40 μM CSR on the proliferation of IL-6/sIL-6R-stimulated RA-FLS decreased by 9%, 15% and 22%, respectively (Fig. 4b). Viability of CSR-untreated RA-FLS^Empty vector^ or CSR-untreated RA-FLS^STAT3C^ was regarded as 100%. These findings indicate that inhibition of the STAT3 signaling contributes to the inhibitory effect of CSR on the cell viability of RA-FLS.

## Discussion

In a previous study, we found that CSR has anti-inflammatory effects in an acute inflammation mouse model [16]. In this study, we demonstrated that CSR inhibits hyperproliferation of RA-FLS. Findings of this and our previous studies suggest that CSR has anti-arthritic potential.

High levels of IL-6 have been observed in joints and blood of RA patients, and serum IL-6 level positively correlates with disease activity [19]. IL-6 exerts its biological activities through IL-6-specific receptor and a signal transducer, gp130 [20]. There are two forms of IL-6-specific receptor: mIL-6R (membrane-bound IL-6R) and sIL-6R. A much higher level of sIL-6R than mIL-6R was detected in synovial fluid and blood of RA patients [21]. Therefore, we used IL-6/sIL-6R-stimulated RA-FLS as the cell model in this study.

When IL‑6 binds to sIL‑6R in RA-FLS, JAK2, an upstream kinase of STAT3, is activated [22], leading to the activation of STAT3. Activated STAT3 forms homodimers and then translocates to the nucleus to regulate the transcription of its target genes [23]. By transcriptionally up-regulating survival genes such as Bcl-2 and Mcl-1, STAT3 signaling promotes RA-FLS hyperproliferation [24, 25]. In this study, it was found that CSR lowers protein levels phospho-JAK2 (Tyr1007/1008), phospho-STAT3 (Tyr705), Bcl-2 and Mcl-1 and decreases STAT3 nuclear localization in IL-6/sIL-6R-stimulated RA-FLS, indicating that CSR inhibits the JAK2/STAT3 pathway in the cell model.

Activation of the JAK2/STAT3 pathway promotes FLS activation/proliferation. FLS proliferation and resistance to apoptosis together with recruitment of other fibroblasts result in synovial hyperplasia, a typical feature of RA [26, 27]. During the progression of RA, hyperplastic synovial membrane is of persistent inflammation, leading to cartilage damage and joint destruction [28]. In the present study, CSR was observed to be able to suppress hyperproliferation of, and induce apoptosis in, IL-6/sIL-6R-stimulated RA-FLS, substantiating that CSR has anti-arthritic potential.

Apart from STAT3, high expression and over-activation of STAT1, another STAT family member that is related to inflammation, can also be observed in the synovium of RA patients [29]. In the RA-FLS model, STAT1 can also be activated by IL-6/sIL-6R stimulation [30]. Suppressing STAT1 expression and inhibiting STAT1 activation have been regarded as approaches for treating RA [30]. Whether CSR exerts anti-RA effects by inhibiting STAT1 signaling remains to be studied.

Hyper-proliferated FLS interact with bone cells, which is another important event involved in joint damage in RA. IL-6/sIL-6R complex induces RA-FLS hyperproliferation through promoting the autocrine of receptor activator of NF-κB ligand (RANKL) [31]. RANKL facilitates the differentiation of osteoclasts from myeloid precursors, leading to bone erosion [32, 33]. To explore the involvement of RANKL in CSR’s anti-RA mechanisms, we will, in the future, investigate whether CSR inhibits RANKL production by suppressing JAK2/STAT3 pathway in IL-6/sIL-6R-stimulated RA-FLS.

## Conclusions

In the present study, we for the first time demonstrated that CSR inhibits hyperproliferation of IL-6/sIL-6R-stimulated RA-FLS, and that inhibition of JAK2/STAT3 signaling is one of the underlying mechanisms. This study provides pharmacological and chemical justifications for the traditional use of CSR-containing herbs in RA treatment, and provides pharmacological groundwork for developing CSR as a novel anti-RA agent. Moreover, this study supports the notion that the JAK2/STAT3 pathway is a target for anti-RA drug discovery.

## Supplementary Information

**Additional file 1. **Effects of CSR on the proliferation of IL-6/sIL-6R-stimulated MIHA and L929 cells. (a) MIHA cell viability. (b) L929 cell viability. In (a) and (b), cells were incubated with indicated concentrations of CSR for 1hr and then stimulated with IL-6/sIL-6R (100 ng/ml each) for 24 hrs. Cell viability was detected using CCK8 assays. Data are expressed as mean ± SD of 3 independent experiments.* *P* < 0.05 *vs.* IL-6/sIL-6R plus CSR solvent-treated group.

**Additional file 2. **Protein levels of STAT3 and phospho-STAT3 (Tyr705) in RA-FLS stimulated with IL-6/sIL-6R (100 ng/ml each)for different durations. β-Actin served as the loading control. Representative immunoblotting results are shown in the left panel. Quantitative results of phospho-STAT3 are shown inthe right panel. Data are expressed as mean ±SD of 3 independent experiments. ** *P* < 0.01 *vs*. 0 min group.

** Additional file 3. **Original blot images of immunoblotting results in Fig. 2b. Representative images of cleaved caspase-3,cleaved caspase-9 and GAPDH are shown. Bands are shown on different films because of different exposure time

**Additional  file 4. **Original blot images of immunoblotting results in Fig. 3. Representative images of JAK2, phospho-JAK2(Tyr1007/1008), STAT3, phospho-STAT3 (Tyr705), STAT3 (cytoplasm), STAT3 (nucleus), Bcl-2, Mcl-1, lamin B1, β-actin and GAPDH are shown. Bands are shown on different films because of different exposure time.

**Additional file 5. **Original blot images of immunoblotting results in Fig. 4. Representative images of Flag, STAT3, phospho-STAT3(Tyr705) and GAPDH are shown. Bands are shown on different films because of different exposure time.

**Additional file 6. **Original blot images of immunoblotting results in Additional file 2. Representative images of STAT3, phospho-STAT3(Tyr705) and β-actin are shown. Bands are shown on different films because of different exposure time.

## Data Availability

All data generated or analyzed during this study are included in this published article, and are available from the corresponding author on reasonable request.

## Abbreviations

Bcl-2: B-cell lymphoma 2
FLS: Fibroblast-like synoviocytes
GFP: Green fluorescent protein
IL-6: Interleukin-6
JAK2: Janus kinase 2
Mcl-1: Myeloid cell leukemia 1
RA: Rheumatoid arthritis
STAT3: Signal transducer and activator of transcription 3
