# Supplementary material for: Chrysoeriol suppresses hyperproliferation of rheumatoid arthritis fibroblast-like synoviocytes and inhibits JAK2/STAT3 signaling
Source: BMC Complement Med Ther. 2022 Mar 16;22:73. doi: 10.1186/s12906-022-03553-w (PMC8928618; doi:10.1186/s12906-022-03553-w)
Supplement: Supplementary file 1 — Additional file 1. Effects of CSR on the proliferation of IL-6/sIL-6R-stimulated MIHA and L929 cells. (a) MIHA cell viability. (b) L929 cell viability. In (a) and (b), cells were incubated with indicated concentrations of CSR for 1hr and then stimulated with IL-6/sIL-6R (100 ng/ml each) for 24 hrs. Cell viability was detected using CCK8 assays. Data are expressed as mean ± SD of 3 independent experiments.* P < 0.05 vs. IL-6/sIL-6R plus CSR solvent-treated group. [file 12906_2022_3553_MOESM1_ESM.docx]

**Additional file 1**


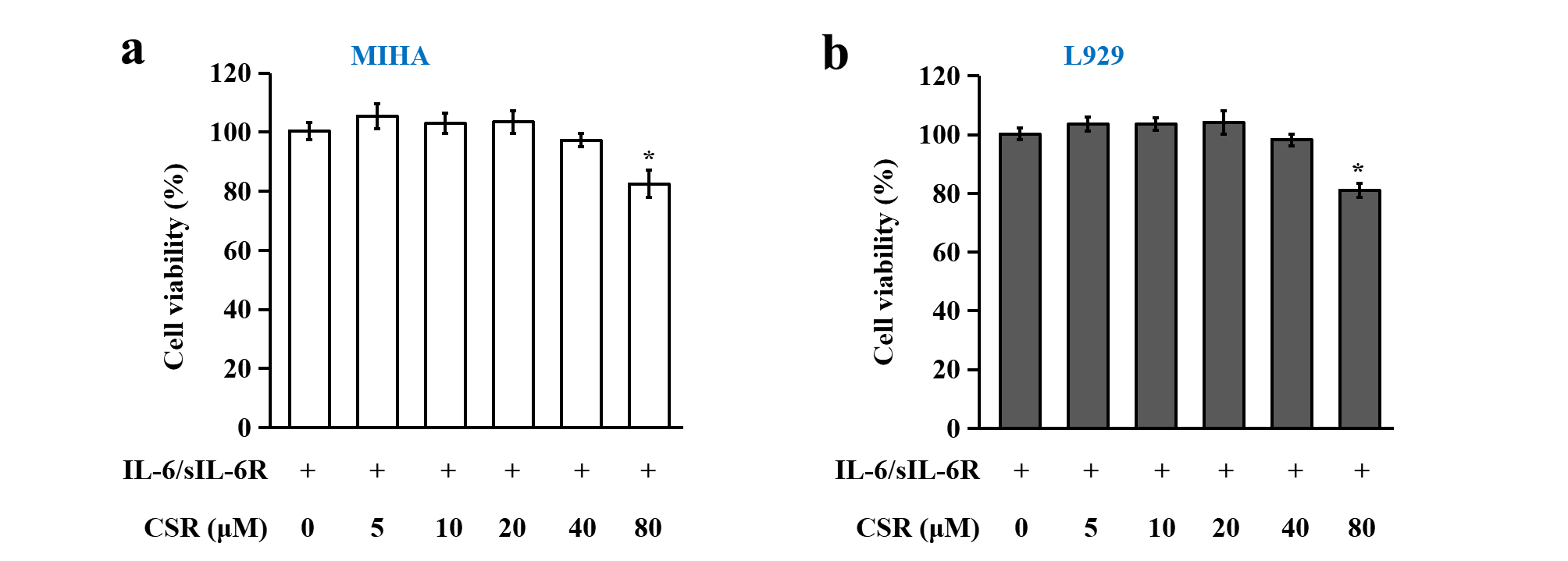


**Additional file 1 .** Effects of CSR on the proliferation of IL-6/sIL-6R-stimulated MIHA and L929 cells. **(a)** MIHA cell viability. **(b)** L929 cell viability. In **(a)** and **(b)**, cells were incubated with indicated concentrations of CSR for 1 hr and then stimulated with IL-6/sIL-6R (100 ng/ml each) for 24 hrs. Cell viability was detected using CCK8 assays. Data are expressed as mean ± SD of 3 independent experiments.* *P* < 0.05 *vs.* IL-6/sIL-6R plus CSR solvent-treated group.
