# Supplementary material for: Chrysoeriol suppresses hyperproliferation of rheumatoid arthritis fibroblast-like synoviocytes and inhibits JAK2/STAT3 signaling
Source: BMC Complement Med Ther. 2022 Mar 16;22:73. doi: 10.1186/s12906-022-03553-w (PMC8928618; doi:10.1186/s12906-022-03553-w)
Supplement: Supplementary file 2 — Additional file 2. Protein levels of STAT3 and phospho-STAT3 (Tyr705) in RA-FLS stimulated with IL-6/sIL-6R (100 ng/ml each)for different durations. β-Actin served as the loading control. Representative immunoblotting results are shown in the left panel. Quantitative results of phospho-STAT3 are shown inthe right panel. Data are expressed as mean ±SD of 3 independent experiments. ** P < 0.01 vs. 0 min group. [file 12906_2022_3553_MOESM2_ESM.docx]

**Additional file 2**


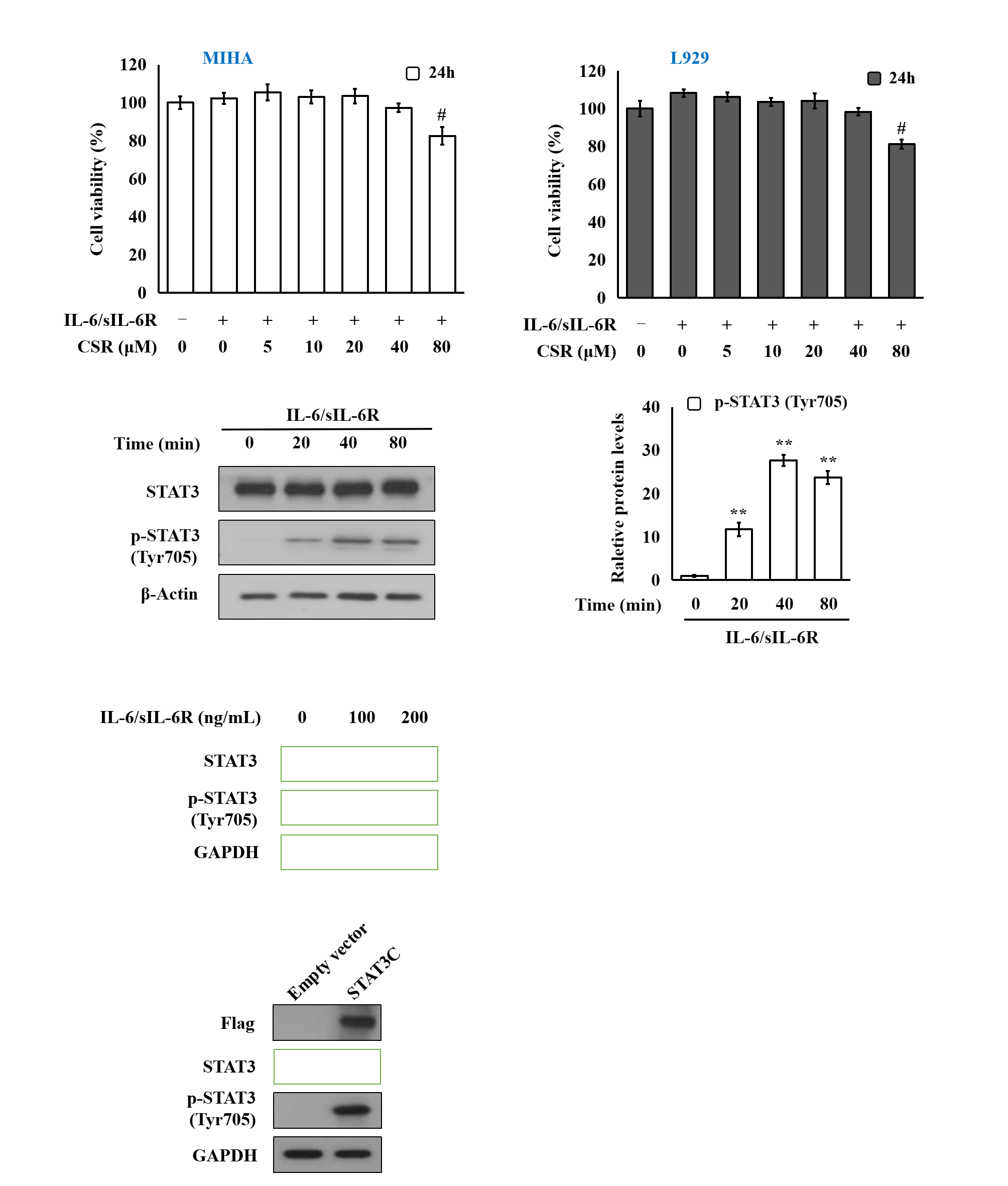


**Additional file 2.** Protein levels of STAT3 and phospho-STAT3 (Tyr705) in RA-FLS stimulated with IL-6/sIL-6R (100 ng/ml each) for different durations. β-Actin served as the loading control. Representative immunoblotting results are shown in the left panel. Quantitative results of phospho-STAT3 are shown in the right panel. Data are expressed as mean ± SD of 3 independent experiments. ** *P* < 0.01 *vs*. 0 min group.
