# Supplementary material for: Chrysoeriol suppresses hyperproliferation of rheumatoid arthritis fibroblast-like synoviocytes and inhibits JAK2/STAT3 signaling
Source: BMC Complement Med Ther. 2022 Mar 16;22:73. doi: 10.1186/s12906-022-03553-w (PMC8928618; doi:10.1186/s12906-022-03553-w)
Supplement: Supplementary file 3 — Additional file 3. Original blot images of immunoblotting results in Fig. 2b. Representative images of cleaved caspase-3,cleaved caspase-9 and GAPDH are shown. Bands are shown on different films because of different exposure time [file 12906_2022_3553_MOESM3_ESM.docx]

**Additional file 3
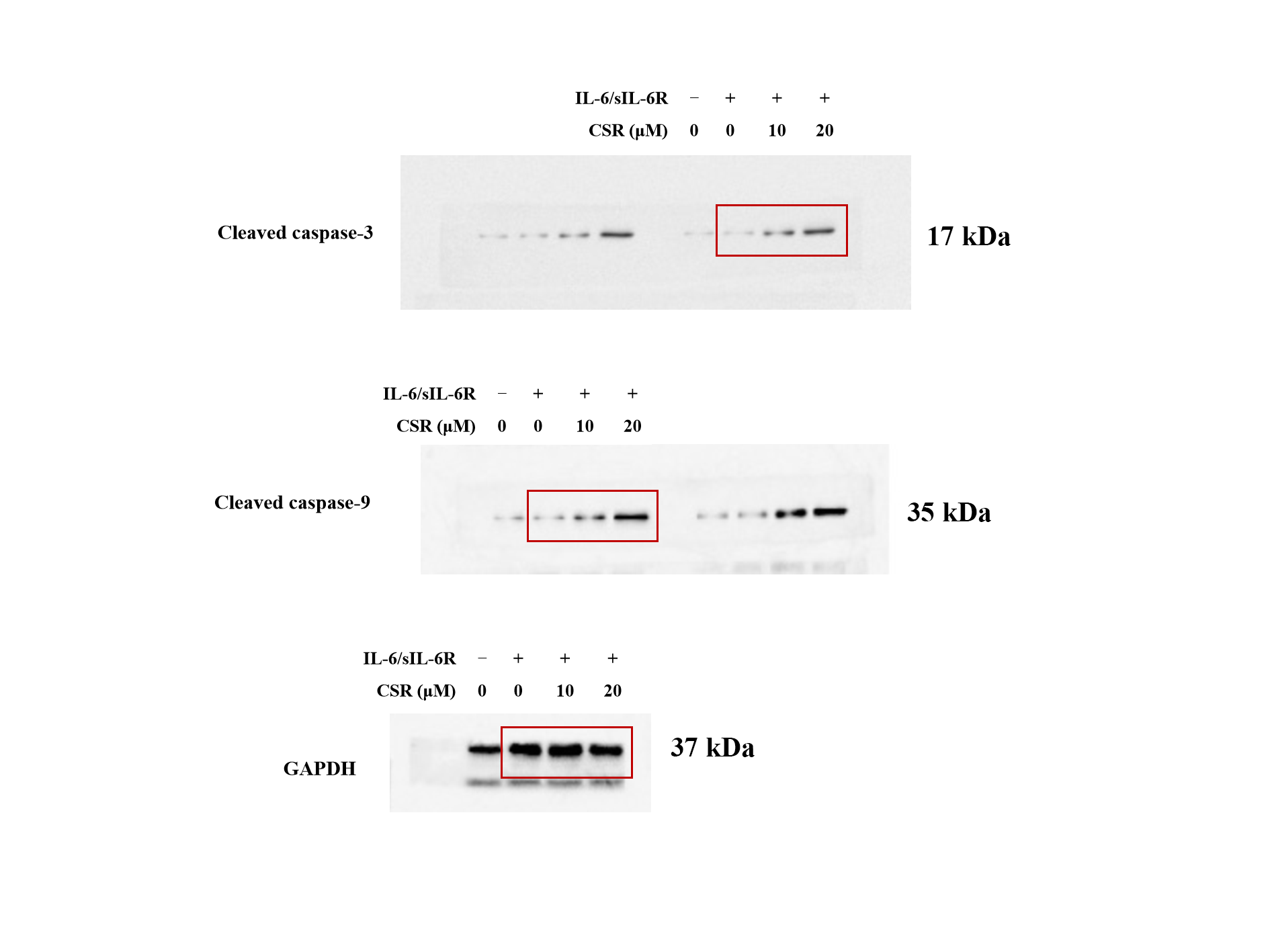
**

**Additional file 3.** Original blot images of immunoblotting results in **Figure 2b**. Representative images of cleaved caspase-3, cleaved caspase-9 and GAPDH are shown. Bands are shown on different films because of different exposure time.
