# Supplementary material for: Chrysoeriol suppresses hyperproliferation of rheumatoid arthritis fibroblast-like synoviocytes and inhibits JAK2/STAT3 signaling
Source: BMC Complement Med Ther. 2022 Mar 16;22:73. doi: 10.1186/s12906-022-03553-w (PMC8928618; doi:10.1186/s12906-022-03553-w)
Supplement: Supplementary file 4 — Additional file 4. Original blot images of immunoblotting results in Fig. 3. Representative images of JAK2, phospho-JAK2(Tyr1007/1008), STAT3, phospho-STAT3 (Tyr705), STAT3 (cytoplasm), STAT3 (nucleus), Bcl-2, Mcl-1, lamin B1, β-actin and GAPDH are shown. Bands are shown on different films because of different exposure time. [file 12906_2022_3553_MOESM4_ESM.docx]

**Additional file 4.**

**
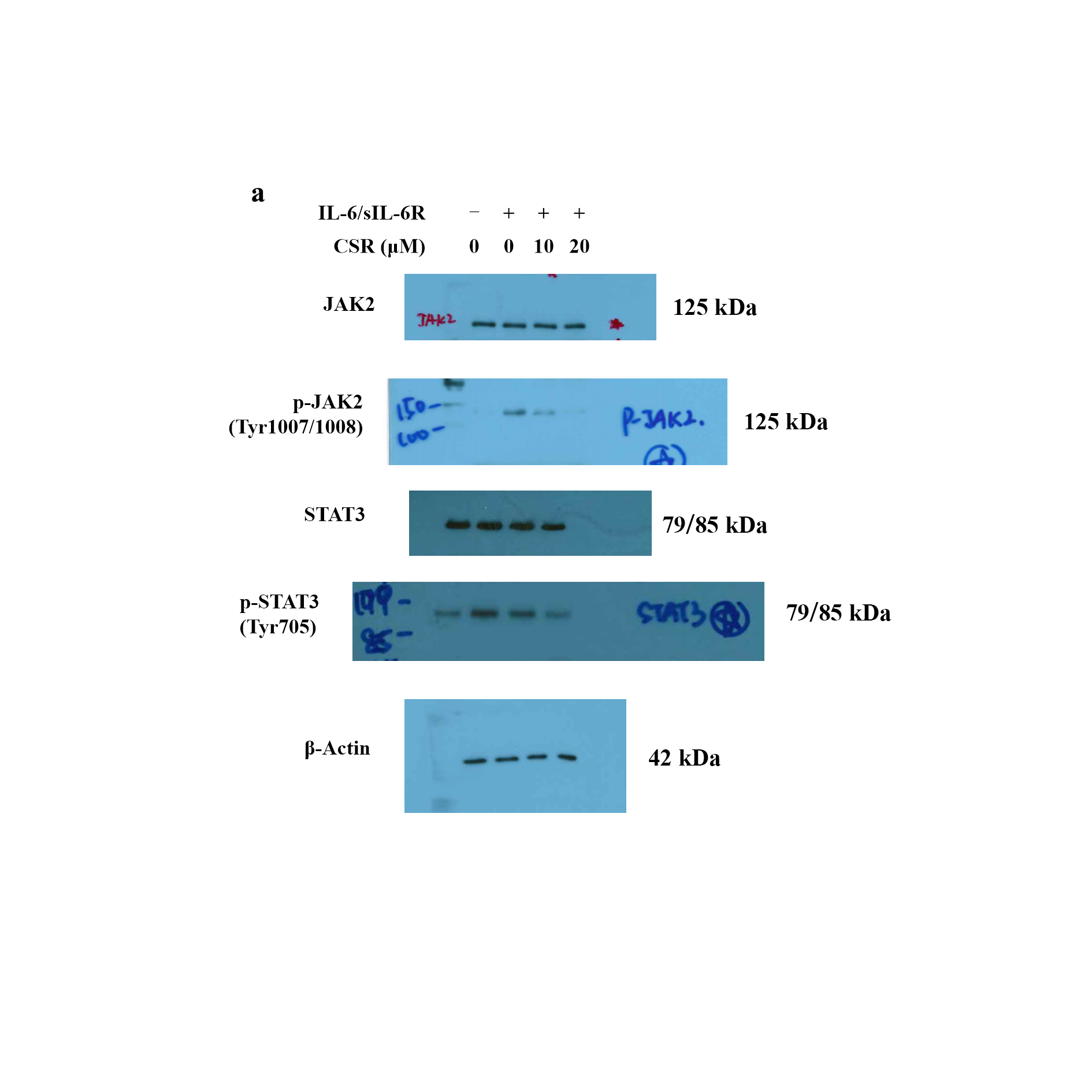
**

**
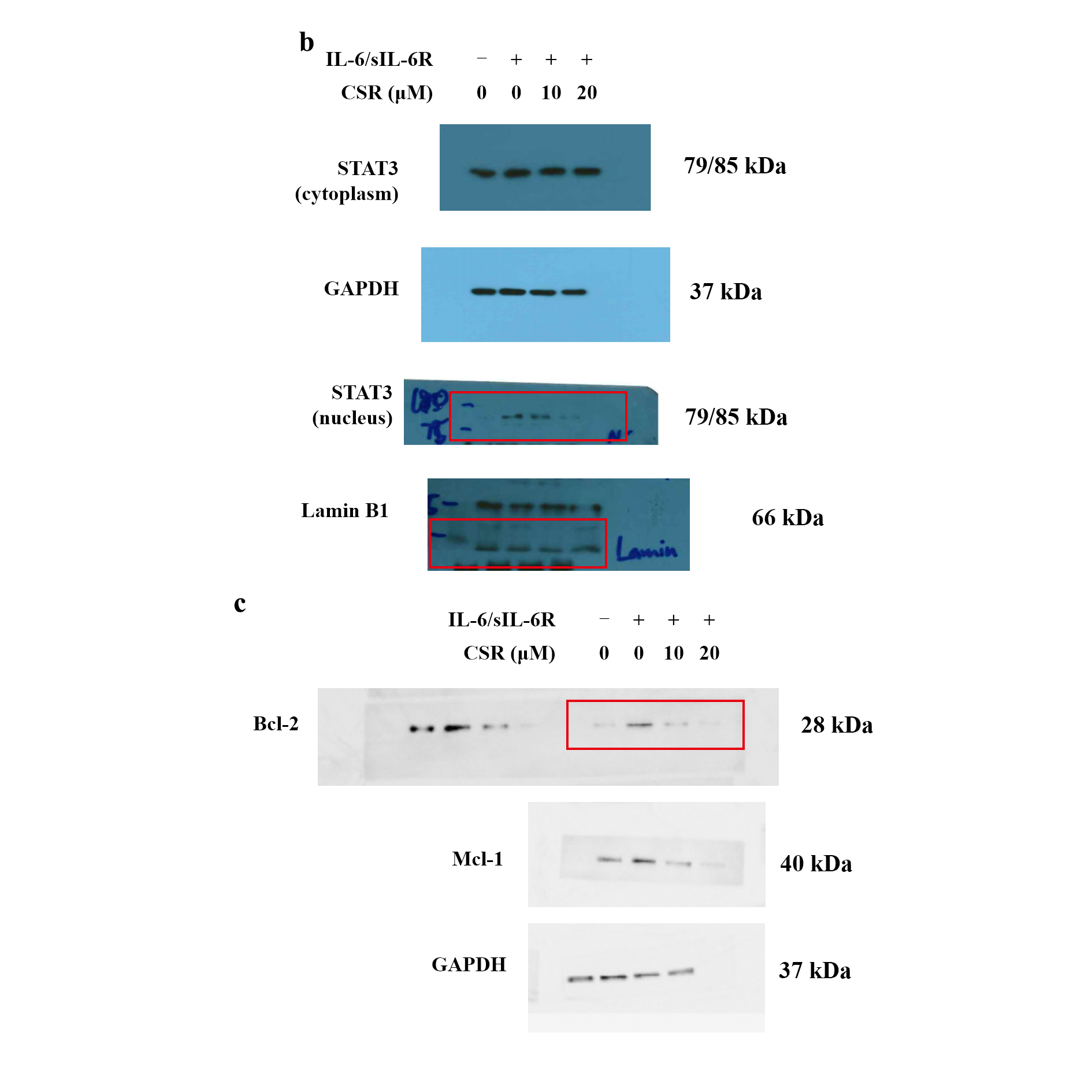
**

**Additional file 4.** Original blot images of immunoblotting results in **Figure 3**. Representative images of JAK2, phospho-JAK2 (Tyr1007/1008), STAT3, phospho-STAT3 (Tyr705), STAT3 (cytoplasm), STAT3 (nucleus), Bcl-2, Mcl-1, lamin B1, β-actin and GAPDH are shown. Bands are shown on different films because of different exposure time.
