# Supplementary material for: Chrysoeriol suppresses hyperproliferation of rheumatoid arthritis fibroblast-like synoviocytes and inhibits JAK2/STAT3 signaling
Source: BMC Complement Med Ther. 2022 Mar 16;22:73. doi: 10.1186/s12906-022-03553-w (PMC8928618; doi:10.1186/s12906-022-03553-w)
Supplement: Supplementary file 5 — Additional file 5. Original blot images of immunoblotting results in Fig. 4. Representative images of Flag, STAT3, phospho-STAT3(Tyr705) and GAPDH are shown. Bands are shown on different films because of different exposure time. [file 12906_2022_3553_MOESM5_ESM.docx]

**Additional file 5.**

**
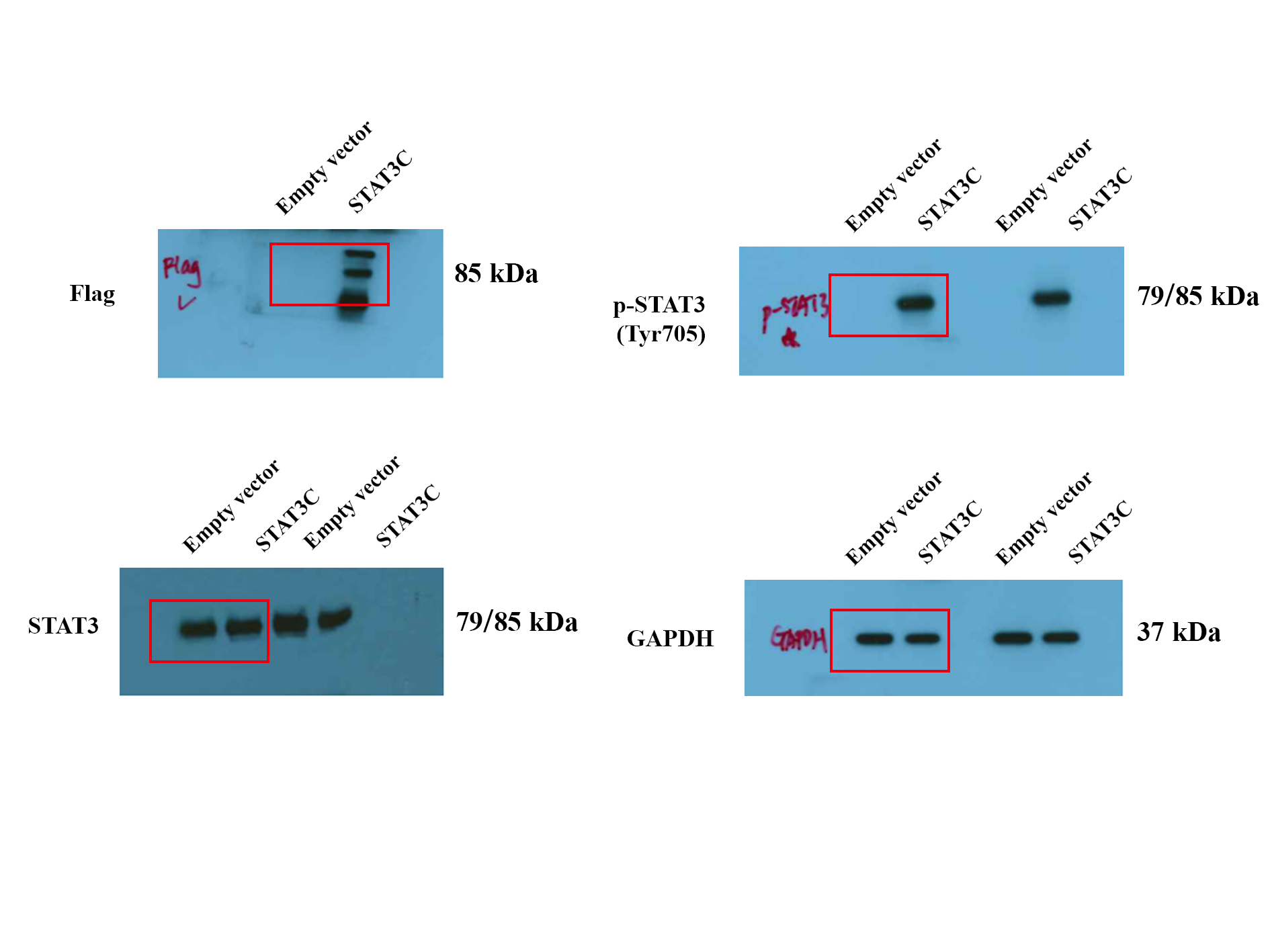
**

**Additional file 5.** Original blot images of immunoblotting results in **Figure 4**. Representative images of Flag, STAT3, phospho-STAT3 (Tyr705) and GAPDH are shown. Bands are shown on different films because of different exposure time.
